# Opposing roles of KIT and ABL1 in the therapeutic response of gastrointestinal stromal tumor (GIST) cells to imatinib mesylate

**DOI:** 10.18632/oncotarget.13882

**Published:** 2016-12-10

**Authors:** Jessica L. Rausch, Sergei Boichuk, Areej A. Ali, Sneha S. Patil, Donna M. Lee, Matthew F. Brown, Kathleen R. Makielski, Ying Liu, Takahiro Taguchi, Shih-Fan Kuan, Anette Duensing

**Affiliations:** ^1^ Cancer Therapeutics Program, University of Pittsburgh Cancer Institute, Hillman Cancer Center, Pittsburgh, PA, USA; ^2^ Department of Anatomy, Kochi Medical School, Nankoku Kochi, Japan; ^3^ Department of Pathology, University of Pittsburgh School of Medicine, Pittsburgh, PA, USA; ^4^ Current address: Department of Pathology, Kazan State Medical University, Kazan, Russia

**Keywords:** gastrointestinal stromal tumor (GIST), imatinib mesylate, KIT, ABL1

## Abstract

Most gastrointestinal stromal tumors (GISTs) are caused by activating mutations of the KIT receptor tyrosine kinase. The small molecule inhibitor imatinib mesylate was initially developed to target the ABL1 kinase, which is constitutively activated through chromosomal translocation in BCR-ABL1-positive chronic myeloid leukemia. Because of cross-reactivity of imatinib against the KIT kinase, the drug is also successfully used for the treatment of GIST. Although inhibition of KIT clearly has a major role in the therapeutic response of GIST to imatinib, the contribution of concomitant inhibition of ABL in this context has never been explored. We show here that ABL1 is expressed in the majority of GISTs, including human GIST cell lines. Using siRNA-mediated knockdown, we demonstrate that depletion of KIT in conjunction with ABL1 – hence mimicking imatinib treatment – leads to reduced apoptosis induction and attenuated inhibition of cellular proliferation when compared to depletion of KIT alone. These results are explained by an increased activity of the AKT survival kinase, which is mediated by the cyclin-dependent kinase CDK2, likely through direct phosphorylation. Our results highlight that distinct inhibitory properties of targeted agents can impede antitumor effects and hence provide insights for rational drug development. Novel KIT-targeted agents to treat GIST should therefore comprise an increased specificity for KIT while at the same time displaying a reduced ability to inhibit ABL1.

## INTRODUCTION

Oncogenic mutations in the *KIT* receptor tyrosine kinase are the tumor-initiating event in the majority of gastrointestinal stromal tumors (GIST) [1]. The resulting constitutive activation of KIT makes GISTs amenable to successful therapy with small molecule KIT kinase inhibitors, such as imatinib mesylate (Gleevec) [2]. Although 85% of patients experience clinical benefit from imatinib therapy, complete remissions are rare and approximately 50% of patients with metastatic or inoperable GIST show disease progression within the first two years of treatment [1, 3]. Dissecting the mechanism of action of imatinib is therefore necessary to develop more effective treatment options.

Imatinib not only inhibits KIT, but also the activity of other tyrosine kinases, most prominently the BCR-ABL1 fusion oncoprotein and the native ABL1 kinase [2, 4]. BCR-ABL1 is generated by the t(9;22) chromosomal translocation and is a hallmark of Philadelphia chromosome-positive (Ph+) chronic myeloid leukemia (CML). Functionally, BCR-ABL1 is characterized by constitutive activation of the ABL1 kinase portion of the protein leading to the activation of several intracellular survival pathways. Many of these signaling cascades, such as RAS/RAF/MEK/MAPK and PI3K/AKT/mTOR, are also activated by oncogenic KIT in GIST [5, 6]. Although BCR-ABL1 does not normally exist in healthy, non-transformed cells or in solid tumors, the non-translocated ABL1 protein is ubiquitously expressed. Hence, inhibition of ABL1 could contribute to the therapeutic effect of imatinib, even when primarily targeting another kinase, such as KIT in GIST.

The non-receptor tyrosine kinase ABL1 was originally identified as the cellular counterpart to the Abelson murine leukemia virus oncogene v-*Abl* [7]. Nevertheless, its physiological functions are still not well understood and may be cell type-specific. Many reports indicate that ABL1 has a role in negatively regulating cellular proliferation and survival. For example, overexpression of wildtype ABL1 in non-malignant fibroblasts resulted in a G1 arrest of the cell division cycle [8]. Moreover, ABL1 mediates a DNA damage-induced cell cycle arrest through direct binding and upregulation of p53 [9]. The role that ABL1 plays in solid tumors is somewhat controversial. Several studies reported overexpression of ABL1 along with evidence that ABL1 has tumor-promoting roles – a finding that would make these tumors amenable to treatment with ABL1 kinase inhibitors [10]. Other reports rather confirm the anti-proliferative role of ABL1. Active ABL1 kinase was shown to inhibit cell viability, proliferation, motility and invasion in breast cancer cells [11]. Similarly, deleting the remaining normal copy of ABL1 in BCR-ABL1^+^ murine leukemia stem cells led to more aggressive disease, enhanced proliferation, inhibition of genotoxic stress-induced apoptosis and increased chromosomal aberrations [12]. Moreover, silencing of ABL1 in mammary epithelial cells led to the induction of epithelial-mesenchymal transition (EMT), a well-studied characteristic of malignant behavior [13, 14]. Notably, nothing is currently known about ABL1 expression in GISTs or the functional impact of ABL1 inhibition in the response to imatinib therapy, to the best of our knowledge.

Herein, we demonstrate that ABL1 is co-expressed with KIT in the vast majority of GISTs. While this finding could point to a potential contribution of ABL1 inhibition to the therapeutic effect of imatinib treatment in these tumors, our results show that it may indeed have the opposite effect. Silencing of KIT in conjunction with ABL1 led to an attenuation of apoptosis induction and attenuation of cell cycle exit when compared to silencing of KIT alone. Importantly, depletion as well as chemical inhibition of ABL1 resulted in increased AKT S473 survival signaling that was mediated by activated CDK2. Taken together, our results indicate that improved future therapies for GIST may be more effective when not targeting ABL1 in conjunction with KIT.

## RESULTS

### KIT and ABL1 are co-expressed in GISTs

**Figure 1:**
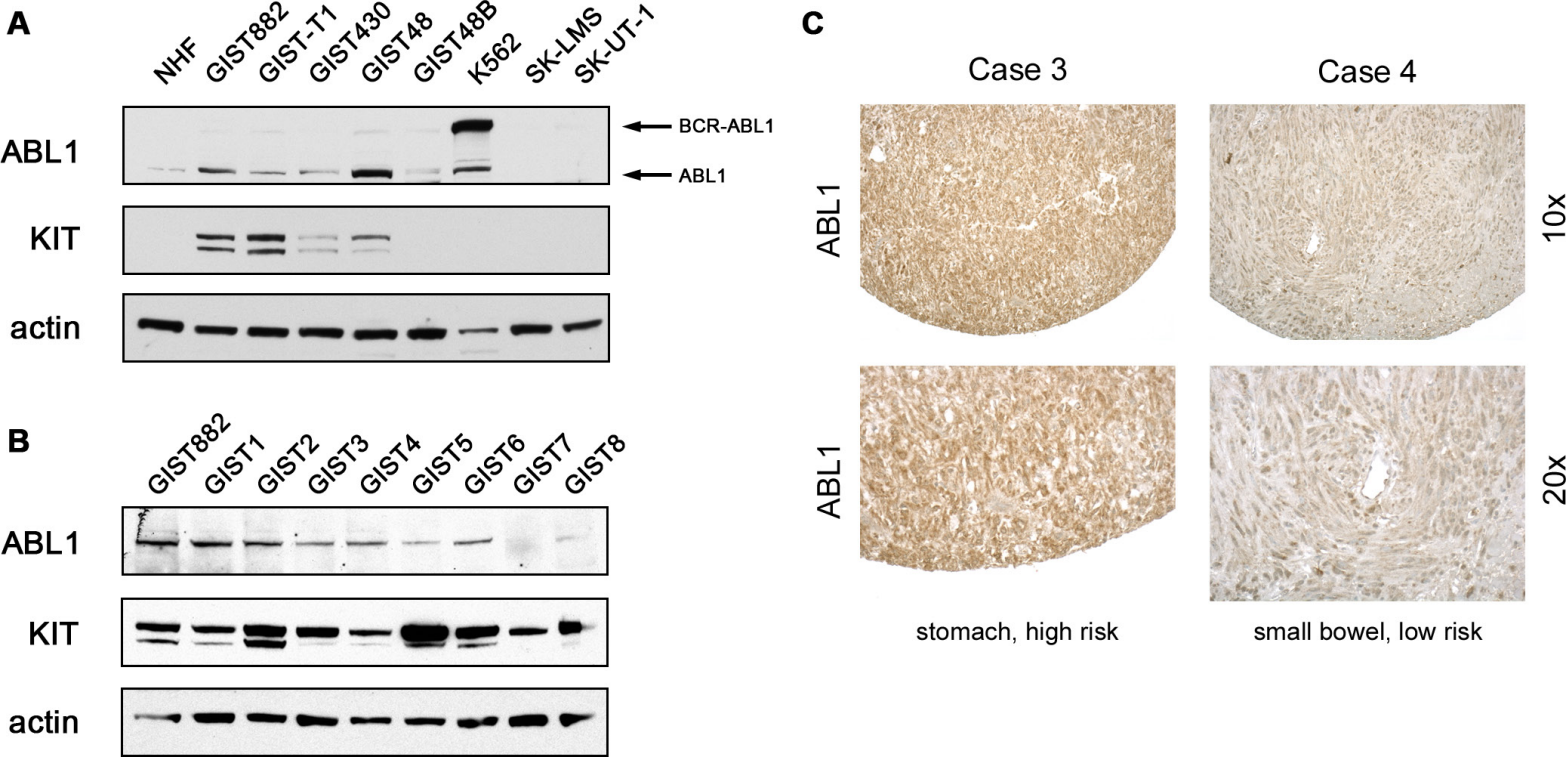
KIT and ABL1 are co-expressed in GIST (**A**) Whole cell lysates of imatinib-sensitive (GIST882, GIST-T1) and imatinib-resistant (GIST430, GIST48, GIST48B) GIST cell lines were immunoblotted for expression of the ABL1 protein. Lysates of normal human fibroblasts (NHF), the BCR-ABL1-expressing CML cell line K562 as well as the human leimyosarcoma cell lines SK-LMS and SK-UT1 were analyzed in comparison. Arrows depict the native ABL1 protein (125 kDa) as well as the BCR-ABL1 fusion protein (210 kDa) detected exclusively in K562 cells. GIST cells show positive expression of KIT, except for GIST48B, which is a known KIT-negative GIST cell line. Actin stain is shown as loading control. (**B**) Whole cell lysates of fresh frozen human GIST samples were immunoblotted for expression of the ABL1 and KIT proteins. GIST882 cell lysates were included to compare expression levels to samples shown in (A). (**C**) ABL1 and KIT expression in primary and metastatic GISTs was assessed by immunohistochemical staining of a tissue microarray (TMA) containing 28 tumors [44]. Examples for high (case 3) and moderate (case 4) ABL1 expression is shown in two GISTs. Top panels, 10× magnification; bottom panels, 20× magnification.

To determine whether ABL1 is expressed in GISTs, we stained imatinib-sensitive (GIST882, GIST-T1) and imatinib-resistant (GIST430, GIST48, GIST48B) GIST cell lines for ABL1 by immunoblotting in comparison to untransformed normal human fibroblasts (NHF; Figure 1A). The BCR-ABL-positive CML cell line K562 served as positive control. All GIST cells expressed ABL1 at levels comparable to the parental ABL1 protein of K562 cells, while NHF expressed ABL1 at a lower level. Interestingly, two leiomyosarcoma cell lines (SK-LMS, SK-UT1) did not express ABL1 at detectable levels. As expected, all GIST cells expressed KIT except GIST48B, which is a KIT-negative derivative of GIST48.

To ascertain that ABL1 expression is also seen in primary GISTs, we first examined eight primary, fresh frozen GIST samples for ABL1 expression by immunoblotting (Figure 1B). Seven of the eight primary tumor samples expressed ABL1, most of them at comparable levels to GIST882. All primary GISTs expressed moderate to high levels of KIT.

Furthermore, we stained a tissue microarray containing 28 GISTs for ABL1 by immunohistochemistry (Figure 1C, Table 1). Individual cores were assessed for ABL1 and KIT expression. Staining intensity was scored as negative (0), weak (0.5–1), moderate (1.5–2) or strong (2.5–3). All assessable samples (27/27; 100%) were positive for ABL1 (Figure 1C, Table 1). The majority were of medium (13) or weak (11) staining intensity with staining found predominately in the cytoplasm. All assessable cores were positive for KIT. One sample was not assessable for both stains, because of absent tumor tissue in the core.

**Table 1:** Patient characteristics

case #	age	gender	location	size [cm]	risk	ABL1	KIT
1	43	F	unknown (m)	mult.	high/metastatic	3	3
2	66	M	small bowel	2.0	none	3	3
3	71	M	stomach	14.0	high	3	0.5
4	50	F	small bowel	4.0	low	2	3
5	65	F	small bowel	5.0	low	2	3
6	44	F	rectum	4.0	high	2	2.5
7	27	M	small bowel	4.5	low	2	2
8	76	M	stomach	5.0	very low	2	2
9	79	M	small bowel	1.5	none	2	2
10	61	M	stomach	21.0	high	2	1.5
11	68	M	unknown (m)	> 10.0	high/metastatic	2	1.5
12	81	M	stomach	6.5	low	2	1
13	92	F	stomach	9.0	low	2	0
14	52	M	small bowel	1.8	none	1.5	2
15	66	M	stomach	13.0	high	1.5	1
16	72	F	stomach	4.0	very low	1.5	0.5
17	43	F	stomach	8.0	low	1	2.5
18	66	M	unknown (m)	mult.	high/metastatic	1	2
19	80	M	small bowel	2.5	low	1	2
20	58	F	stomach	13.0	intermediate	1	1.5
21	78	M	stomach	1.5	none	1	1
22	64	M	stomach	2.0	none	1	1
23	75	F	stomach	4.0	very low	1	0.5
24	82	M	colon	6.0	high	1	0
25	69	F	stomach	3.5	very low	0.5	2
26	32	F	stomach	0.7	none	0.5	1
27	57	M	retroperitoneum	20.0	high/metastatic	0.5	0.5
28	69	M	stomach	1.0	n/a	n/a	n/a

Cases are grouped according to their ABL1 expression levels (from high to low). No correlation with age, gender, tumor location, tumor size or risk for recurrence (assessed according to [46]) were seen. There was a weak correlation between ABL1 and KIT expression levels (*r* = 0.354). (m), metastatic; mult., multiple tumor nodules present; n/a, core not assessable.

Patient characteristics are shown in Table 1. There were 17 males and 11 females with an average age of 63.7 years. Most tumors were located in the stomach (15/28), while seven tumors were from the small bowel. The remaining tumors were in the colon or rectum (2/28), retroperitoneum (1/28) or metastatic at time of diagnosis (3/28). There was no correlation between ABL1 expression levels and tumor location, risk of recurrence (*p* > 0.05; Fisher's Exact Probability test), age (*p* > 0.05; ANOVA) or gender (*p* > 0.05; Fisher's Exact Probability test). There was a weak correlation between ABL1 and KIT expression levels (correlation coefficient *r* = 0.354).

Taken together, ABL1 is co-expressed with KIT in the majority of GISTs. Because imatinib inhibits both, inhibition of ABL1 could contribute to the therapeutic effect of imatinib treatment of GIST.

### Depletion of ABL1 counteracts knockdown of KIT

Having shown that GISTs express both KIT and ABL1, we aimed to assess their relative contributions to GIST cell proliferation and/or survival. siRNA-mediated knockdown of either KIT or ABL1 in GIST882 cells was used to mimic single inhibition of either of these proteins. In analogy, a combined knockdown of both KIT and ABL1 mimicked imatinib treatment.

**Figure 2:**
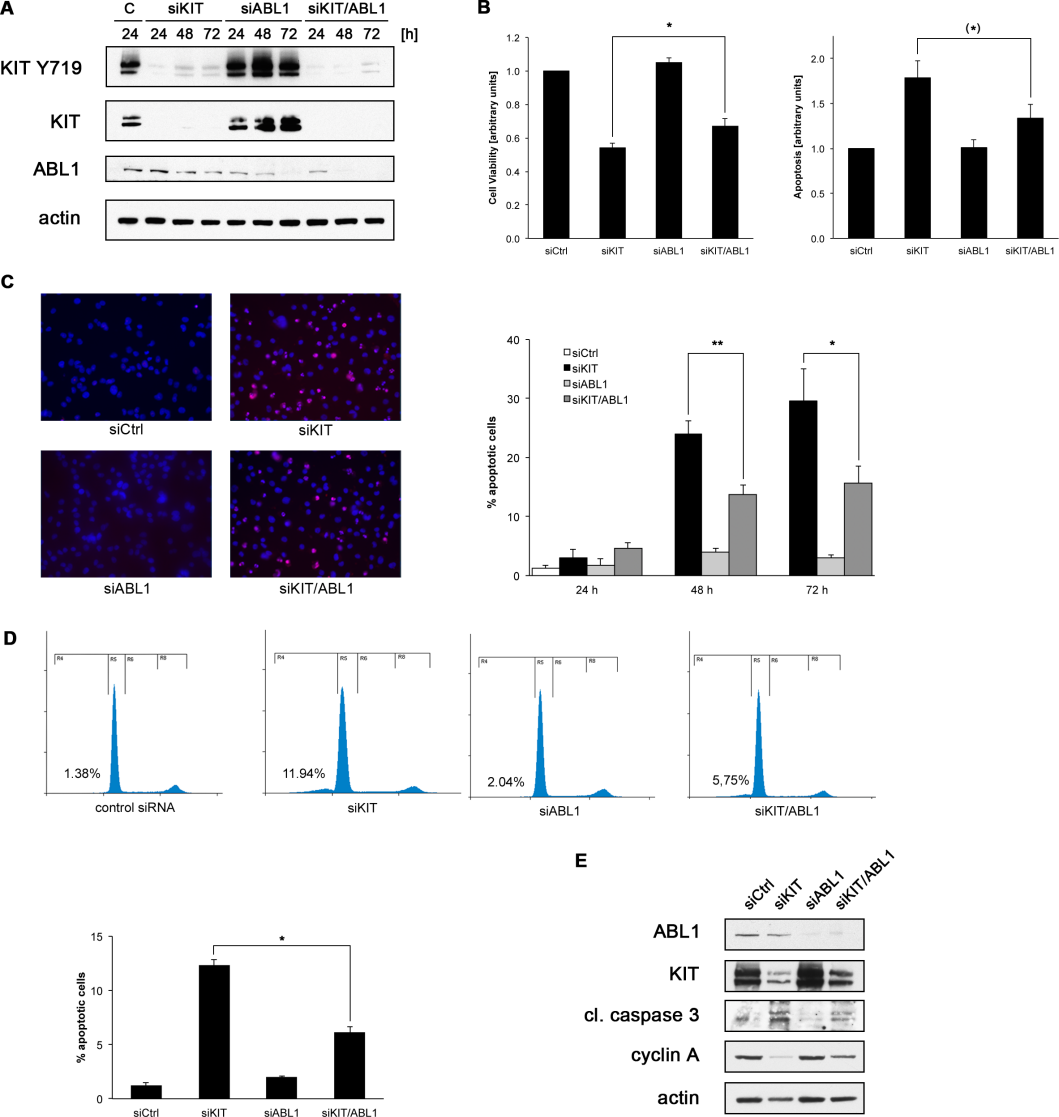
Co-depletion of KIT and ABL1 attenuates the effects of KIT knock-down (**A**) GIST882 cells were transfected with non-targeted siRNA control sequences (“C”) or small interfering RNA (siRNA) sequences targeting KIT and ABL1 either alone or in combination. Whole cell lysates obtained 24, 48 or 72 hours after transfection were immunoblotted for expression levels of phosphorylated (Y719) and total KIT as well as ABL1. (**B**) GIST882 cells were transfected as described in (A). Cell viability (left panel) and apoptosis (caspase 3/7 activity; right panel) were assessed 72 hours post transfection using luminescence-based assays. Results were normalized to transfection with non-targeted siRNA controls. Error bars represent standard error of the mean (SEM). *p < 0.02; (*)*p* < 0.08 (one-tailed *t*-test). (**C**) GIST882 cells were transfected as described in (A) and the percentage of apoptotic cells was determined using the TUNEL assay (red), left panels. Nuclei are stained with DAPI. Quantitation of apoptotic cells transfected with non-targeted siRNA control sequences (white bar) or siRNA sequences targeting KIT (black bars), ABL1 (light grey bars) or KIT and ABL1 in combination (dark grey bars) at the indicated time points, right panel. **p < 0.02; *p < 0.05 (one-tailed *t*-test). (**D**) GIST882 cells were transfected as described in (A) and their cell cycle profile was determined by flow cytometry (top panels). Bottom panel shows quantitation of the percentage of cells detected in the sub-G1 population (apoptotic cells). Error bars represent standard deviation. *p < 0.007. A representative experiment is shown. (**E**) GIST882 cells were transfected as described in (A) and whole cell lysates (72 hours after transfection) were immunoblotted for ABL1 and KIT to document appropriate knockdowns. Blots were further probed for markers of apoptosis (cleaved caspase 3) and cell cycle activity (cyclin A).

siRNA-mediated knockdown of KIT and ABL1 occurred within 24 to 48 hours of transfection in both single and combination experiments (Figure 2A). As expected, siRNA-mediated knockdown of KIT led to a significantly reduced cellular proliferation (Figure 2B, left panel; *p* < 0.0001) and increased apoptosis (Figure 2B, right panel; *p* < 0.01) when compared to transfection with non-targeting siRNA control in luminescence-based assays. By contrast, siRNA-mediated knockdown of ABL1 showed little to no effect on GIST882 proliferation or apoptosis (Figure 2B; *p* > 0.05).

To our surprise, depletion of KIT and ABL1 in combination did not lead to an increased effect when compared to depletion of KIT alone, but rather to a significantly attenuated reduction of cellular proliferation (*p* < 0.02) and a strong trend towards a reduced induction of apoptosis (*p* < 0.08). Similar results were seen in a time course experiment over 72 hours when using the TUNEL assay for readout (Figure 2C). At 48 and 72 hours after transfection, siRNA-mediated knockdown of KIT and ABL1 in combination led to a significantly attenuated induction of apoptosis when compared to knockdown of KIT alone (*p* < 0.02 and *p* < 0.05, respectively).

Moreover, we could show that combined silencing of KIT and ABL1 resulted in a significantly attenuated increase of the sub-G1 fraction detected by flow cytometry when compared to silencing of KIT alone (Figure 2D; *p* < 0.007).

The above results were corroborated by biochemical analyses (Figure 2E). Depletion of KIT and ABL1 in combination led to an attenuated induction of caspase 3 cleavage when compared to depletion of KIT alone. Similarly, reduction of cyclin A levels were attenuated under these conditions.

Taken together, these results indicate that loss of ABL1 in addition to KIT attenuates the pro-apoptotic and anti-proliferative effect of KIT depletion in GIST cells and could thus be disadvantageous in the therapeutic setting.

### Inhibition of ABL1 leads to activation of AKT

Having shown that loss of ABL1 in addition to KIT may be disadvantageous in the therapeutic setting of GIST, we set out to dissect the molecular mechanism of this phenomenon.

**Figure 3:**
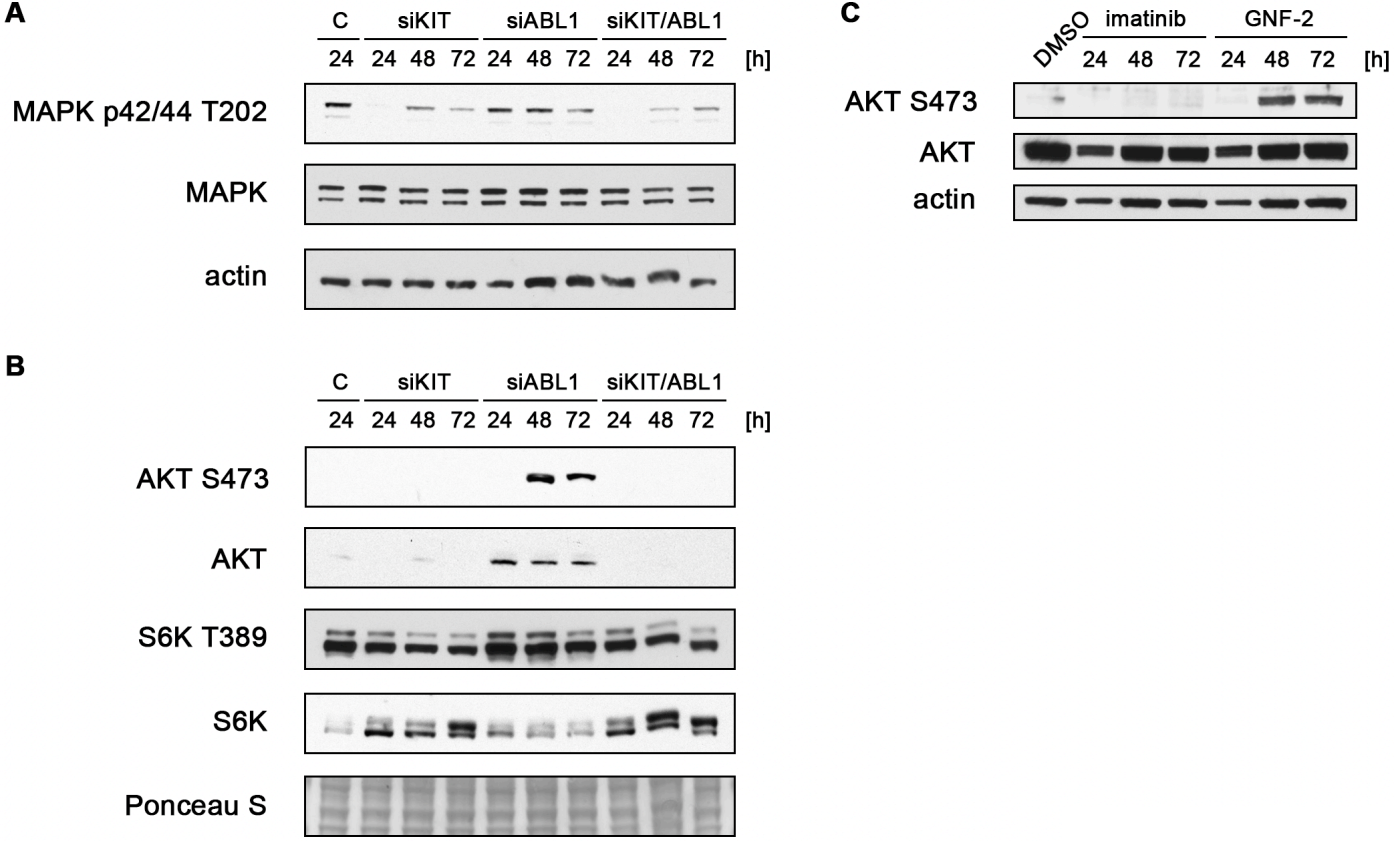
ABL1 knockdown and chemical inhibition of ABL1 induce activation of AKT (**A**, **B**) GIST882 cells were transfected with non-targeted siRNA control sequences (“C”) or siRNA sequences targeting KIT and ABL1 either alone or in combination. Whole cell lysates obtained 24, 48 or 72 hours after transfection were immunoblotted for expression levels of phosphorylated (T202) and total MAPK p42/44 (A) as well as phosphorylated (S473)/total AKT and phosphorylated (T389)/total S6K (B). (**C**) GIST882 cells were treated with DMSO control, the KIT/ABL1 inhibitor imatinib mesylate or the allosteric ABL1 inhibitor GNF-2. Whole cell lysates were immunoblotted for phosphorylated (S473) and total AKT.

We first analyzed whole cell lysates of GIST882 cells after siRNA-mediated transfection of KIT and ABL1 for known signaling mediators downstream of KIT by immunoblotting (Figure 3A). As expected, knockdown of KIT led to inhibition of MAPK signaling as assessed by reduced MAPK p42/44 phosphorylation at T202 (Figure 3A). This effect was present to a similar extent after combined silencing of KIT and ABL1, but not seen after depletion of ABL1 alone. Similarly, the AKT–S6 kinase (S6K) axis was inhibited after siRNA-mediated knockdown of KIT (Supplementary Figure S1, Figure 3B). To our surprise, however, silencing of ABL1 led to a substantial increase in S473-phosphorylated AKT resulting in increased levels of T389-phosphorylated S6 kinase.

To test whether the induction of AKT activation can also be induced by chemical inhibition of ABL1, we treated GIST882 cells with the ABL1-specific allosteric inhibitor GNF-2, a compound that does not inhibit KIT (Figure 3C) [15]. GNF-2 is a 4,6-disubstituted pyrimidine that specifically inhibits BCR-ABL1 and ABL1 by binding to the myristoyl binding cleft, an allosteric binding site distant from the active site of the kinase [15, 16]. We could show that GNF-2 treatment indeed resulted in a substantial increase in AKT phosphorylation at S473.

Together, we show that loss or chemical inhibition of ABL1 in GIST cells leads to an increase in AKT survival signaling. These results can therefore explain the attenuated effect on cellular proliferation and apoptosis after combined KIT/ABL1 knockdown when compared to depletion of KIT alone. Hence, the known ABL1 inhibitory function of imatinib in addition to inhibition of KIT may counteract its therapeutic effect in GIST.

### AKT activation after loss of ABL1 is not due to increased PDK1 activity or impaired AKT dephosphorylation

While an increase in AKT S473 activation provides an explanation for the attenuated induction of apoptosis and increased cellular proliferation after functional loss of ABL1, a direct link between ABL1 and AKT signaling has not been described yet to the best of our knowledge.

**Figure 4:**
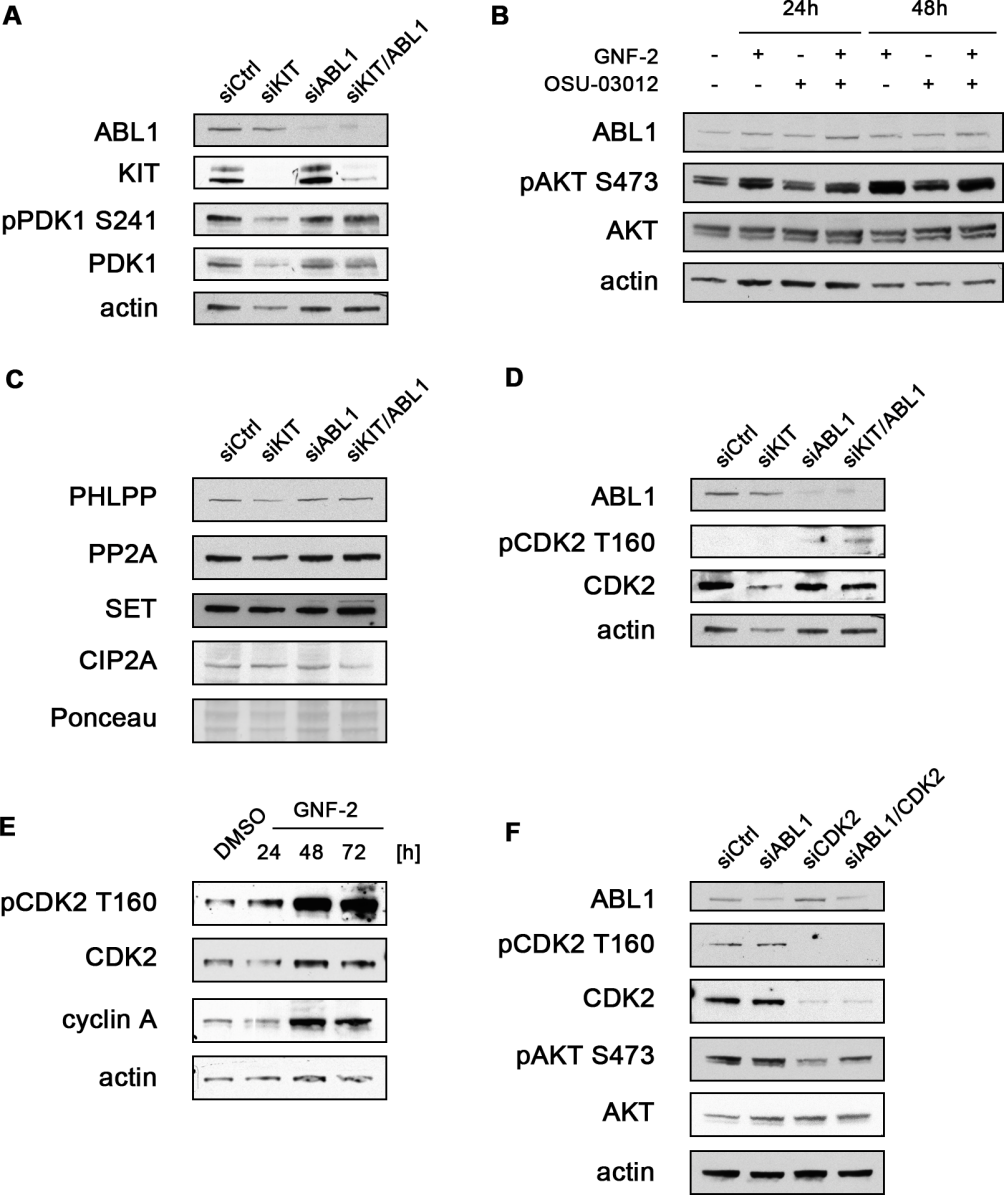
AKT activation after siRNA-mediated knockdown or chemical inhibition of ABL1 is mediated by CDK2 (**A**) GIST882 cells were transfected with non-targeted siRNA control or siRNA targeting KIT and ABL1 either alone or in combination. Whole cell lysates (72 hours) were immunoblotted for KIT and ABL1 expression levels to confirm appropriate knockdowns. The blot was further probed for phosphorylated (S241) and total PDK1. (**B**) GIST882 cells were treated with DMSO or the allosteric ABL1 inhibitor GNF-2 and the PDK1 inhibitor OSU-03012 either alone or in combination. Whole cell lysates were immunoblotted for expression levels of ABL1, phosphorylated (S473) and AKT. (**C**) GIST882 cells were transfected as described in (A). Whole cell lysates were immunoblotted for known regulators of AKT dephosphorylation (PHLPP, PP2A, SET, CIP2A). Total protein Ponceau S stain is shown as a loading control. (**D**) GIST882 cells were transfected as described in (A). Whole cell lysates were immunoblotted for ABL1 expression as well as phosphorylated (T160) and total CDK2. (**E**) GIST882 cells were treated with DMSO control or the allosteric ABL1 inhibitor GNF-2 for the indicated times. Whole cell lysates were immunoblotted for expression levels of phosphorylated (T160) and total CDK2 as well as cyclin A. (**F**) GIST882 cells were transfected with non-targeted siRNA control sequences or siRNA sequences targeting ABL1 and CDK2 either alone or in combination. Whole cell lysates (72 hours) were immunoblotted for ABL1 and CDK2 expression levels to confirm appropriate knockdowns. The blot was further probed for phosphorylated (T160) and total CDK2 as well as phosphorylated (S473) and total AKT.

To dissect the pathways leading to increased AKT activation after siRNA-mediated knockdown or chemical inhibition of ABL1, we first evaluated activation of its upstream activating kinase PDK1. However, no changes in PDK1 phosphorylation levels were seen after siRNA-mediated knockdown of either ABL1 alone or in combination with KIT when compared to depletion of KIT alone (Figure 4A). Hence, a different mechanism is likely responsible for phosphorylating AKT after depletion of ABL1.

To corroborate these results, we inhibited PDK1 activity using the small molecule PDK1 inhibitor OSU-03012 alone and in combination with the ABL1 inhibitor GNF-2 (Figure 4B). While chemical inhibition of PDK1 alone led to a slight reduction of AKT S473 phosphorylation, it did not attenuate AKT activation when used in combination with the ABL1 inhibitor GNF-2. These results further indicate that PDK1 is not involved in mediating AKT activation after functional loss of ABL1.

We next tested whether increased AKT phosphorylation after depletion of ABL1 could be a result of impaired dephosphorylation of AKT. However, no changes in expression levels of phosphatases that are either directly (PHLPP, PP2A) or indirectly (SET, CIP2A) involved in AKT dephosphorylation were detected after ABL1 knockdown (Figure 4C). Together, these findings provide an indication that a novel mechanism may lead to AKT activation after functional loss of ABL1.

### CDK2 mediates activation of AKT after loss of ABL1

A recent study reported that the cyclin-dependent kinase CDK2 has the capability to directly phosphorylate AKT thereby enabling its full activation [17]. CDK2 acts in concert with cyclin A2, the predominant mammalian cyclin A isoform, facilitating entry into S phase [18, 19]. We have shown above (Figure 2E) that siRNA-mediated knockdown of ABL1 leads to increased expression levels of cyclin A2 and increased cellular proliferation. Therefore, we tested whether CDK2 could be directly responsible for leading to increased AKT phosphorylation levels after silencing or chemical inhibition of ABL1.

We first tested whether siRNA-mediated knockdown of ABL1 leads to increased CDK2 activity. Indeed, silencing of ABL1 alone or in combination with siRNA-mediated knockdown of KIT resulted in increased levels of CDK2 that is phosphorylated at T160 (Figure 4D). Similarly, chemical inhibition of ABL1 with GNF-2 also caused a significant increase in CDK2 activation (Figure 4E). Together, these findings suggest that increased proliferative activity after functional inhibition of ABL1 is due to an increased activity of CDK2/cyclin A2 complexes.

To test whether CDK2 could be directly responsible for the increased levels of AKT phosphorylation after functional loss of ABL1, we reduced CDK2 expression levels through siRNA-mediated knockdown alone or in combination with ABL1. As shown in Figure 4F, silencing of CDK2 led to a significant reduction in AKT S473 activation when compared to transfection with non-targeting control siRNA. Importantly, however, knockdown of CDK2 in conjunction with ABL1 attenuated the increase in AKT S473 activation induced by knockdown of ABL1 alone. Our results thereby indicate that CDK2 plays a key role in eliciting a pro-proliferative signal after functional loss of ABL1: first, by directly stimulating entry into the cell division cycle (S phase) together with its partner cyclin A2, and second, by inducing AKT phosphorylation – thereby further potentiating a pro-survival effect.

Taken together, a reduced ABL1 inhibitory capacity is likely beneficial for the effectiveness of KIT inhibitors in the treatment of GIST.

### Sunitinib and regorafenib are weak ABL1 inhibitors

The multi-kinase inhibitors sunitinib and regorafenib are currently the only FDA-approved second- and third-line therapies for imatinib-resistant GIST, respectively [20]. While their efficacy in the advanced setting is in part due to the ability to inhibit *KIT* secondary mutations and likely also their broader kinase inhibitory spectrum, a reduced ability to inhibit ABL1 could be of additional benefit, as stated above.

**Figure 5:**
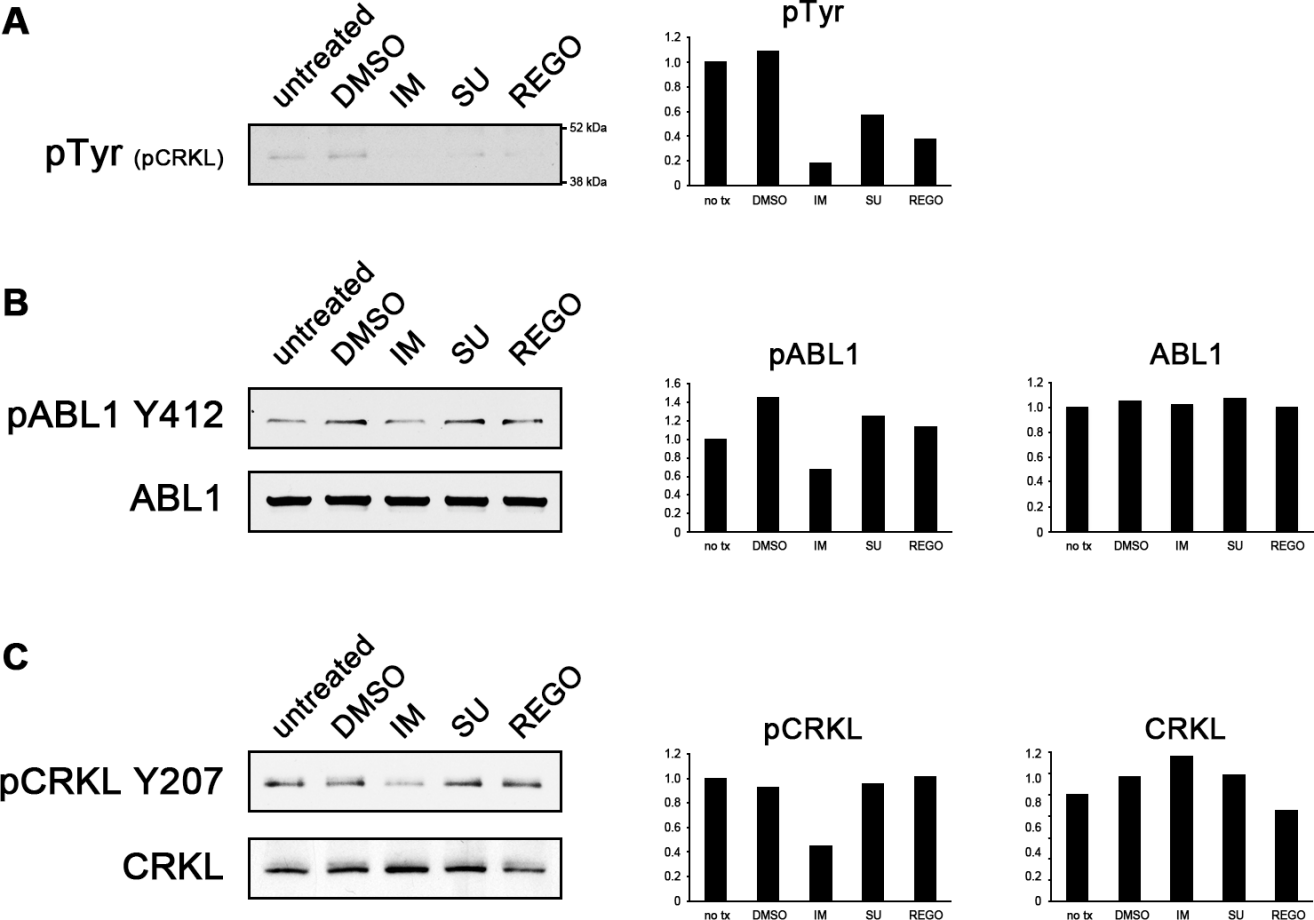
Imatinib inhibits ABL1 kinase activity more effectively than sunitinib or regorafenib (**A**–**C**) A non-radioactive *in vitro* ABL1 kinase assay was performed using recombinant CRKL protein as a substrate. Reactions were performed in the presence or absence of imatinib (IM), sunitinib (SU) or regorafenib (REGO). Staining for global kinase phosphorylation (A), ABL1 phosphorylation (B) and CRKL phosphorylation (C) shows that imatinib inhibits ABL1 whereas sunitinib and regorafenib are substantially less effective ABL1 inhibitors. In (A), the bands likely depict phosphorylated CRKL, a ~40 kDa protein. Band intensity was measured using LI-COR Image Studio Lite (right panels) and values were normalized to the untreated sample. No tx, no treatment.

We therefore compared the ability of sunitinib and regorafenib to inhibit ABL1 kinase activity and phosphorylation of its downstream effector CRKL with that of imatinib. CRKL is a well-established substrate of ABL1 [21]. However, it has also been shown to be downstream of KIT [22]. We thus chose to perform an *in vitro* kinase assay to circumvent any unspecific phosphorylation that could be due to KIT activity in GIST cells (Figure 5). We were indeed able to show that imatinib inhibits ABL1 phosphorylation (at Y412) as well as ABL1's ability to phosphorylate its downstream target CRKL to a significantly greater extent than sunitinib or regorafenib (Figure 5). Together, these results corroborate the notion that the reduced ability of sunitinib and regorafenib to inhibit ABL1 contributes to their effectiveness in the treatment of imatinib-resistant GIST.

In summary, our study identifies ABL1 inhibition as an adverse off-target effect of KIT kinase inhibitors used to treat GIST, which counteracts their efficacy. It is thus desirable to reduce the ABL1 inhibitory capacity when developing new KIT inhibitors in order to identify more effective therapies for GIST patients.

## DISCUSSION

The majority of gastrointestinal stromal tumors is characterized by oncogenically activating mutations of the *KIT* receptor tyrosine kinase and can hence successfully be treated with the KIT inhibitor imatinib mesylate. However, it is well known that imatinib also strongly inhibits the oncogenic fusion protein BCR-ABL1 as well as the intracellular protein kinase ABL1. BCR-ABL1, generated by the t(9;22) chromosomal translocation, is almost exclusively expressed in CML. By contrast, the native, non-translocated ABL1 kinase is a ubiquitously expressed protein. It is therefore conceivable that inhibition of ABL1 could contribute to the therapeutic effect of imatinib GIST. In the present study, we could show that ABL1 is indeed co-expressed with KIT in the majority of GISTs, including human GIST cell line models. However, co-depletion of KIT and ABL1 using siRNA-mediated knockdown – thus mimicking treatment with the KIT/ABL1 inhibitor imatinib – led to attenuated pro-apoptotic and anti-proliferative responses when compared to depletion of KIT alone. This effect was mediated, at least in part, by a novel mechanism that involves direct phosphorylation of the AKT survival kinase by the cyclin-dependent kinase CDK2.

Our results may seem surprising, because the ABL1 kinase is often viewed as an oncoprotein that is driving proliferation and the evolution of a malignant phenotype. However, most of this notion stems from studies of the BCR-ABL1 fusion oncogene. By contrast, the native ABL1 protein kinase is a negative regulator of the cell division cycle [8]. Early studies have shown that overexpression of wildtype ABL1 leads to cell cycle arrest in G1 [8]. In addition, ABL1 is necessary for mediating stress response and growth arrest as well as for mediating apoptosis in response to DNA-damaging agents [17, 23, 24]. Importantly, a recent study by Skorski et al. showed that deletion of the remaining normal copy of ABL1 in BCR-ABL1^+^ murine leukemia stem cells led to more aggressive disease, enhanced proliferation, inhibition of genotoxic stress-induced apoptosis and increased chromosomal aberrations [12]. These findings are in line with our results showing that depletion or chemical inhibition of ABL1 leads to increased cellular proliferation and that a combined depletion of KIT and ABL1 attenuates the growth inhibitory and pro-apoptotic response of single depletion of KIT. Other studies have shown that ABL1 is required for the release of cytochrome c in the oxidative stress response. ER stress leads to translocation of ABL1 from ER to mitochondria and to subsequent cytochrome c release and apoptosis [25]. Therefore, ABL1 has a role in targeting pro-apoptotic stress signals to the mitochondria. A study by Ito et al. could show that only wildtype, but not *Abl1*^−/−^ cells responded to the induction of ER stress with the induction of apoptosis [25]. Similarly, another study described that treatment of NIH 3T3 fibroblasts with the ABL1 inhibitor GNF-2 led to localization of n-myristoylated ABL1 to ER thereby presumably diminishing the pool that can translocate to the mitochondria to induce apoptosis [16]. Interestingly, imatinib has been shown to induce apoptosis in GIST cells in part via the induction of ER stress [26]. It is therefore tempting to speculate that treating GIST cells with a KIT inhibitor that does not simultaneously inhibit ABL1 could further enhance this response.

We show here that depletion as well as inhibition of ABL1 leads to activation of the AKT pro-survival pathway. These results explain why co-depletion of KIT and ABL1 has an attenuated effect on apoptosis induction and inhibition of proliferation when compared to depletion of KIT alone. Interestingly, dissecting the mechanism of action leading to AKT S473 phosphorylation after functional loss of ABL1 showed that this effect was neither mediated by PDK1, the upstream activating kinase of AKT, nor by signaling cascades that regulate desphosphorylation of AKT. Instead, we found that AKT phosphorylation was mediated through the cyclin-dependent kinase CDK2. It is well-documented that CDK2 is negatively regulated by ABL1 [9]. ABL1 is activated in response to DNA damage, and downregulates CDK2 activity, which in turn leads to a reversible growth arrest in G1 [9]. Consequently, a significantly higher proportion of cells are found to be in S phase after irradiation when comparing *Abl*^−/−^ mouse embryonic fibroblasts (MEFs) with wildtype MEFs [9]. Our results are completely in line with these findings, as we could show that depletion as well as chemical inhibition of ABL1 leads to increased CDK2 activity (Figure 4). Notably, CDK2 has recently been shown to directly phosphorylate AKT at S477 and T479 [17]. This phosphorylation at the extreme carboxy-terminal region of AKT was shown to facilitate AKT S473 phosphorylation leading to full activation of AKT. When we depleted CDK2 in conjunction with ABL1 we noted an attenuated increase of AKT phosphorylation at S473 when compared to depletion of ABL1 alone indicating that CDK2 is indeed responsible for increased AKT S473 activity after functional inhibition of ABL1. Together, these findings may point to a potentially beneficial effect of combining a CDK inhibitor with imatinib. Notably, a clinical trial testing the efficacy and safety of the CDK4/6 inhibitor PD-0332991 in patients with advanced GIST is currently ongoing (CYCLIGIST, NCT01907607) [27].

A recent report by Corbin et al. addressed a similar topic as our study [28]. While we were interested in the impact that ABL1 inhibition has in the therapeutic effect of imatinib in GIST, these authors investigated the impact of KIT inhibition on the therapeutic effect of BCR-ABL1 inhibition by imatinib in CML [28]. The majority of CML cells not only express KIT, but KIT has been implicated in the pathogenesis of CML. Interestingly, the study by Corbin et al. shows that KIT inhibition is indeed necessary in addition to inhibition of BCR-ABL1 for maximal suppression of mature CML progenitor cells (CD34^+^/CD38^+^). Similar results were noted by Belloc et al. when testing primary CD34^+^ CML cells [29]. These findings may seem to contradict our study. However, similar to us, Corbin et al. describe an induction of AKT S473 phosphorylation after specific inhibition of BCR-ABL1 by PPY-A, a compound that does not inhibit KIT [30]. This effect was most pronounced after concomitant stimulation of the KIT receptor by stem cell factor. Importantly, the authors also noted an increased proliferative response under these conditions. PPY-A also strongly inhibits the native ABL1 kinase [30]. It is therefore very well possible that AKT S473 activation is mediated by the same mechanism as in our study: a direct phosphorylation by activated CDK2 after inhibition of native ABL1. Because AKT is a signaling mediator downstream of KIT, its activation is downregulated through KIT inhibition – thereby explaining why inhibition of KIT in addition to BCR-ABL1 is needed for a full therapeutic response in CML.

The results of our study support the notion of ABL1's role as a tumor suppressor and “anti-target” in GIST [31, 32]. While certain off-target effects of anticancer drugs can be beneficial, inhibition of an “anti-target” is an off-target effect that negatively affects its effectiveness. ABL1 may have a dual role as an “anti-target” in GIST. As discussed above, inhibition of ABL1 likely reduces the anti-tumor effectiveness of sole KIT inhibition in GIST. These results are supported by the clinical observation that tyrosine kinase inhibitors with no ABL1 inhibitory component are more effective in GIST than those that do. For example, sunitinib and regorafenib, the FDA-approved second- and third-line treatments for imatinib-resistant GIST do not significantly inhibit ABL1 compared to imatinib as shown in an *in vitro* kinase assay (Figure 5). The same is true for sorafenib, which is very similar to regorafenib on the molecular level [20]. On the other hand, nilotinib and dasatinib – both strong ABL1, but comparatively lesser KIT inhibitors– have proven less effective than imatinib in GIST clinical trials [33, 34]. Consequently, several efforts are currently underway to develop highly specific KIT inhibitors [35, 36]. A further negative effect of ABL1 inhibition in GIST may be the introduction of unnecessary toxicity. ABL1 has been implicated in mediating cardiotoxic adverse effects in several KIT/ABL1 inhibitors, such as imatinib [37]. As mentioned above, however, not all off-target effects are adversary. As discussed earlier, the KIT inhibitory component of imatinib was shown to be beneficial for the treatment of CML [28]. There is also a consensus that the broader inhibitory spectrum of sunitinib and regorafenib that includes VEGFR inhibition is a contributing factor to these compounds’ effectiveness in GIST. In addition, it has been reported that inhibition of PDGFRA/B may be beneficial for the therapeutic response in GIST. A recent study showed that PDGFRA/B inhibition further reduced MAPK signaling and potentiated the downregulation/degradation of ETV1 when compared to KIT inhibition alone [38].

Taken together, our study not only adds an important piece to the puzzle of the mechanism of action of imatinib in GIST. More importantly, we contribute a guide to future drug development for GIST, as it seems prudent to reduce the ABL1 inhibitory capacity when developing new KIT inhibitors for the therapy of GIST.

## MATERIALS AND METHODS

### Cell culture, inhibitor treatments and transfections

The imatinib-sensitive human GIST cell lines GIST882 (a generous gift from Jonathan A. Fletcher, Brigham and Women's Hospital, Harvard Medical School, Boston, MA) and GIST-T1 [39] were derived from untreated, metastatic GISTs and were maintained as previously described [40]. Imatinib-resistant GIST cell lines GIST430, GIST48, and GIST48B (also provided by J.A. Fletcher) were derived from human GISTs that developed clinical resistance to imatinib therapy and were grown as previously described [41]. GIST48B cells are derived from GIST48 cells, with which they share the *KIT* mutational status, but show no detectable KIT protein expression [42]. All cell lines were obtained directly from the original investigator and not further authenticated.

K562, a BCR-ABL1-positive chronic myelogenous leukeumia cell line, as well as the SK-UT1, SK-LMS leiomyosarcoma cell lines were obtained from ATCC and not further authenticated. Normal human dermal fibroblasts were obtained from Lonza and not passaged for longer than six months. All cells were maintained according to manufacturer's recommendations.

For inhibitor treatments, cells were incubated in imatinib mesylate (1 μM in DMSO; LC Laboratories), the allosteric ABL1 inhibitor GNF-2 (1 μM in DMSO; Sigma-Aldrich), the PDK1 inhibitor OSU-03012 (10 μM in DMSO; Apexbio Technology) or mock-treated with 0.1% DMSO for up to 72 hours, as indicated.

For small interfering RNA (siRNA) experiments, pooled synthetic RNA duplexes (siGENOME SmartPool; Dharmacon) were used to reduce protein expression of KIT, ABL1 or CDK2 alone or in combination. Briefly, cells were trypsinized, and 3 × 10^6^ cells were transfected with 5 μl of 10 μM annealed RNA duplexes using nucleofection (Amaxa/Lonza). Cells were then transferred to 35 mm tissue culture dishes with 2 ml RPMI1640 free of antibiotics and incubated for up to 72 hours. Knock-down efficiency was monitored by immunoblotting.

### GIST patients and tissue microarray

Fresh GIST tissue was collected from patients undergoing tumor resection at the University of Pittsburgh School of Medicine Presbyterian Hospital (IRB#0509050). The tissue was fresh frozen and protein extracts were prepared as described previously [43].

For the tissue microarray, a total of 28 cores from primary and metastatic GISTs and seven controls (liver) were collected from the archives of the Department of Pathology at the University of Pittsburgh School of Medicine (IRB#0509050) [44].

### Immunological and cell staining methods

Protein lysates of cells growing as a monolayer were prepared as described previously [40]. Protein concentrations were determined by the Bradford assay (Biorad). 30 μg of protein were loaded on a 4–12% Bis-Tris gel (Invitrogen) and blotted onto a nitrocellulose membrane.

Immunohistochemistry of paraffin-embedded sections was performed as described previously [45]. Antigen-retrieval consisted of microwaving in 0.01 M citrate buffer (pH 6.0) for 10 min. Immunoperoxidase-based detection was performed using the Histostain-Plus 3rd Gen IHC Detection Kit (Invitrogen/Thermo Fisher Scientific) according to manufacturer's recommendations. Cells were analyzed using an Olympus AX70 epifluorescence microscope equipped with a SpotRT digital camera.

Primary antibodies used for immunoblotting and immunohistochemistry were ABL1, CDK2, pTyr (all Santa Cruz), actin (Sigma), pABL1 Y412, pAKT S473, AKT, pCDK2 T160, cleaved caspase 3, pCRKL Y207, CRKL, pKIT Y719, pMAPK p42/44 T202, pPDK1 S241, PDK1, PP2A, pS6K T389, S6K (all Cell Signaling Technologies), CIP2A, PHLPP, SET (all Bethyl Laboratories), cyclin A (Novocastra), KIT (DakoCytomation) and MAPK (Invitrogen/Thermo Fisher Scientific).

### *In vitro* apoptosis and proliferation assays

Apoptosis and cell viability studies were performed using the Caspase-Glo and CellTiter-Glo luminescence-based assays (Promega) [41]. Cells were plated in 96-well flat-bottomed plates (Perkin Elmer) after transfection and incubated for 48 hours (Caspase-Glo) or 72 hours (CellTiter-Glo). Luminescence was measured with a BioTek Synergy 2 Luminometer (BioTek). Data were normalized to the cells transfected with non-targeting control siRNA.

### TUNEL assay

Apoptotic cells were detected using the TUNEL assay (Roche) according to manufacturer's recommendations as described previously [41].

### Flow cytometry

Cell cycle analysis was performed by measuring the amount of propidium iodide (PI)-labeled DNA in ethanol-fixed cells. In brief, cells were harvested by trypsinization, washed twice with pre-chilled PBS (containing 1% FBS), and fixed with ice-cold 70% ethanol. After the fixation step, cells were washed with PBS/1% FBS, resuspended in propidium iodide (PI; Sigma-Aldrich)/RNase staining solution (50 μg/ml PI, 10 mM Tris pH 7.5, 5 mM MgCl_2_, 10 μg/ml RNase) and incubated at 37°C in the dark for 30 min. The analysis was performed in the University of Pittsburgh Cancer Institute Flow Cytometry Core Facility using a Gallios Flow Cytometer (Beckman Coulter) and the Kaluza 5 acquisition software.

### *In vitro* kinase assay

A non-radioactive *in vitro* kinase assay was performed using 150 ng each of recombinant active ABL1 (SignalChem) and recombinant CRKL protein (SignalChem) as a substrate in 20 μl kinase buffer (25 mM Tris HCl, pH 7.4; 10 mM MgCl_2_) containing 100 μM ATP. The reaction was incubated for 30 minutes at 30°C, stopped by adding loading buffer with β-Mercaptoethanol and heating the sample at 95°C. Proteins were separated by SDS-gel electrophoresis, and phosphorylation of ABL1 and CRKL was assessed by immunoblotting for phosphorylated tyrosine (pY99, Santa Cruz), phospho-ABL1 Y412 and phospho-CRKL Y207 (both antibodies from Cell Signaling). Antibodies against total ABL1 and total CRKL were from Santa Cruz and Cell Signaling, respectively. Band intensity was measured using LI-COR Image Studio Lite (LI-COR).

### Statistical analysis

Statistical significance was assessed using the Student's *t*-test, ChiSquare test, Fisher Exact Probability test or ANOVA analysis wherever applicable (http://vassarstats.net; http://department.obg.cuhk.edu.hk/researchsupport/statmenu.asp). *P*-values of *p* ≤ 0.05 were considered statistically significant.

## SUPPLEMENTARY MATERIALS FIGURES


