# Supplementary material for: Opposing roles of KIT and ABL1 in the therapeutic response of gastrointestinal stromal tumor (GIST) cells to imatinib mesylate
Source: Oncotarget. 2016 Dec 10;8(3):4471–83. doi: 10.18632/oncotarget.13882 (PMC5354847; doi:10.18632/oncotarget.13882)
Supplement: Supplementary file 1 [file oncotarget-08-4471-s001.pdf]

Opposing roles of KIT and ABL1 in the therapeutic response of gastrointestinal stromal tumor (GIST) cells to imatinib mesylate

Supplementary Materials

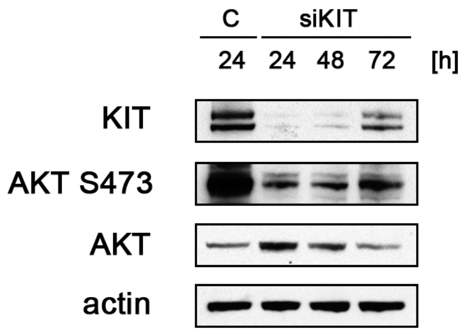

Supplementary Figure S1: KIT knockdown leads to inhibition of AKT S473. GIST882 cells were transfected with non-targeted siRNA control sequences (“C”) or siRNA sequences targeting KIT. Whole cell lysates obtained 24, 48 or 72 hours after transfection were immunoblotted for expression levels of KIT as well as phosphorylated (S473) as well as total AKT. Actin stain is shown as a loading control.
